# Influence of the Location of a Decision Cue on the Dynamics of Pupillary Light Response

**DOI:** 10.3389/fnhum.2021.755383

**Published:** 2022-01-26

**Authors:** Pragya Pandey, Supriya Ray

**Affiliations:** Centre of Behavioural and Cognitive Sciences, University of Allahabad, Prayagraj, India

**Keywords:** eye movement, pupillometry, decision-making, model simulation, human, countermanding

## Abstract

The pupils of the eyes reflexively constrict in light and dilate in dark to optimize retinal illumination. Non-visual cognitive factors, like attention, arousal, decision-making, etc., also influence pupillary light response (PLR). During passive viewing, the eccentricity of a stimulus modulates the pupillary aperture size driven by spatially weighted corneal flux density (CFD), which is the product of luminance and the area of the stimulus. Whether the scope of attention also influences PLR remains unclear. In this study, we contrasted the pupil dynamics between diffused and focused attentional conditions during decision-making, while the global CFD remained the same in the two conditions. A population of 20 healthy humans participated in a pair of forced choice tasks. They distributed attention to the peripheral decision cue in one task, and concentrated at the center in the other to select the target from four alternatives for gaze orientation. The location of this cue did not influence participants’ reaction time (RT). However, the magnitude of constriction was significantly less in the task that warranted attention to be deployed at the center than on the periphery. We observed similar pupil dynamics when participants either elicited or canceled a saccadic eye movement, which ruled out pre-saccadic obligatory attentional orientation contributing to PLR. We further addressed how the location of attentional deployment might have influenced PLR. We simulated a biomechanical model of PLR with visual stimulation of different strengths as inputs corresponding to the two attentional conditions. In this homeomorphic model, the computational characteristic of each element was derived from the physiological and/or mechanical properties of the corresponding biological element. The simulation of this model successfully mimicked the observed data. In contrast to common belief that the global ambient luminosity drives pupillary response, the results of our study suggest that the effective CFD (eCFD) determined *via* the luminance multiplied by the size of the stimulus at the location of deployed attention in the visual space is critical for the magnitude of pupillary constriction.

## Introduction

Pupillary light response (PLR) maintains retinal illumination when the intensity of ambient light changes. The pupils of the eyes reflexively and consensually constrict in light and dilate in dark. PLR is not completely reflexive; many non-visual cognitive factors, including attention (e.g., Binda et al., 2013; Naber et al., 2013), saccadic eye movement preparation (Jainta et al., 2011; Mathôt et al., 2015; Wang et al., 2018; Pandey and Ray, 2021; Wang and Munoz, 2021), decision-making (e.g., de Gee et al., 2014; Sheng et al., 2020), and even subliminal stimuli can influence PLR (Laeng et al., 2012; Einhäuser, 2017; Mathôt, 2018). On the other hand, pupil size too can influence cognition; for instance, movement planning (Cherng et al., 2020) and esthetic appraisal evaluation (Liao et al., 2021).

A plethora of evidence shows that sustained spatial attention modulate PLR. Covert orientation of attention without orienting gaze to the darker side of a display exhibited a larger pupil size in comparison to when attention oriented to the brighter side (Mathôt et al., 2013). A change in the peripheral luminance, while fixation was maintained at the center of the display, affected PLR more when this change in the light intensity happened at the attended hemifield than at the unattended hemifield (Binda and Murray, 2015). Temporal attention (Wierda et al., 2012), feature-based selective attention (Einhäuser et al., 2008; Binda et al., 2014; Turi et al., 2018; but see Hupé et al., 2008), and obligatory pre-saccadic attention (Mathôt et al., 2015) also influence PLR.

A non-uniform distribution of retinal photoreceptors and pupillary aperture in combination limit the burden of visual processing of entire scene in high resolution, which could otherwise be overwhelming (Geisler and Banks, 1995; Strasburger et al., 2011). While densely packed cone receptors in the fovea results in rapidly decreasing visual acuity toward the periphery, pupillary aperture regulates an influx of light on the retina. Thus, PLR plays a key role in perception from the earliest stage of vision (Ebitz and Moore, 2019). In fact, as the luminance changes in the phototopic range, PLR results in pupillary aperture giving the maximum spatial resolution (Campbell and Gregory, 1960). However, peripheral objects may still be selected for further processing by widening the scope of attention (Castiello and Umiltà, 1990; Balz and Hock, 1997; Goto et al., 2001).

Several studies tested the influence of the eccentricity of visual stimulus and attentional breadth on pupil size, notwithstanding, their relationships remained inconclusive. Daniels et al. (2012) presented a pair of concentric circular arrays of stimuli, one at the parafoveal (proximal to central fixation) and the other at the perifoveal (distal from central fixation) visual space. Rhythmic constriction and dilation of the pupil were observed when participants’ attention periodically and transiently switched between the central and peripheral array of stimuli keeping the gaze fixed at the center of the display. Pupil diameter decreased and increased when attentional breadth was narrow and was broad. However, no difference in the average pupil diameter was observed between narrowly focused and broadly diffused attention sustained for the duration of an entire trial. Similarly, in another study, the pupils transiently constricted more when the scope of attention was narrow to identify the local components in Navon figures (i.e., a large figure composed of small figures) in comparison to when it was broad to identify the global components, but the mean pupillary constriction did not vary with the attentional state (DiCriscio et al., 2018). In this line, attentional enhancement in PLR was found to be independent of whether attention was spread around the peripheral stimulus or concentrated at the center of the stimulus (Binda and Murray, 2015). Hu et al. (2020) also did not find the influence of attentional breadth on the magnitude of pupillary constriction. A larger pupil size was found to be associated with attention shifted to distal stimuli than proximal stimuli in a task that warranted a shift of attention to report the number of pre-specified objects embedded in a set of stimuli placed at one of the three different eccentricities (Brocher et al., 2018). Ivanov et al. (2019) conducted an experiment wherein the participants reported the orientation of Gabor patches at any of the three possible eccentricities on both left and right sides of the display while maintaining fixation at the center. The locations of the patches were pre-cued. The eccentricity of the bilateral cues where participants presumably allocated attention did not influence pre-target tonic pupil size (Mathôt, 2020). In these studies, participants’ attentional breadth changed while they were either passively viewing the stimuli, or counting items, or performing a detection task. Whether the scope of covert selective attention that enables us to discriminate the target from distractor(s) (Johnston and Dark, 1986; Corbetta and Shulman, 2002) influences PLR remains unknown.

In primate’s brain, the frontal eye field (FEF) is an area that not only contributes to covert orientation of attention for the discrimination of the target from distractors (Wardak et al., 2006; Murthy et al., 2009) and the goal-directed saccadic eye movement (Hanes and Schall, 1996; Ray et al., 2009; Sendhilnathan et al., 2021), FEF neurons also send projections to the parasympathetic PLR pathway responsible for pupillary constriction (Wang and Munoz, 2015; Ebitz and Moore, 2017). Recently, we found that the dynamics of visually evoked pupil dynamics might act as a proxy of the FEF activity during target selection and saccade planning (Pandey and Ray, 2021). In the current study, we tested whether pupillary response could be distinguished between the deployment of attention either focused at the center or distributed to the periphery to select the target from alternatives for gaze orientation. We recruited healthy young participants to perform a pair of decision-making tasks. In one task, a decision cue was placed just outside the central vision (parafoveal), and in another, it was placed further away from the center in the periphery of a display (perifoveal). Participants discriminated the target from distractors and rapidly oriented gaze to the target to indicate their choice. The quality of the stimuli and timing of visual events were identical, and the eccentricity of the saccade target was the same between the tasks. We dissociated covert selective attention from obligatory pre-saccadic attention by introducing a stop-signal sporadically. We observed that the dynamics of pupillary constriction was influenced by the eccentricity of a decision cue (i.e., attentional breadth) independent of the elicitation of a saccade.

In the phototopic range, pupil size decreases with increasing corneal flux density (CFD) that depends on the product of luminance and the adapting field size of the stimulus (Stanley and Davies, 1995; Park and McAnany, 2015). During passive viewing of the stimulus of steady luminance, the estimated pupil size based on CFD weighted by a two-dimensional (2D) Gaussian function with the peak of the function at the location of gaze fixation (i.e., center of the display screen) closely followed the measured pupil size (Zhang et al., 2019). Here, we further tested whether CFD filtered by the scope of attention drives pupillary response. To this end, we simulated a homeomorphic biomechanical model of pupillary muscle plants, which was successfully used to estimate the sympathetic and parasympathetic activity in the PLR pathway for pupillary flash response (Usui and Hirata, 1995; Yamaji et al., 2000). We used two different levels of visual stimulation as inputs to the model for the two task conditions corresponding to the focused and diffused attention to the decision cues—higher for the latter as the peripheral cue was larger in size than the central cue. Note that the colors of the decision cues, either central or peripheral, were luminance matched, but the area occupied by the peripheral cue was larger than the area occupied by the central cue. Mimicry of the empirical data using model simulation suggests that the effective CFD (eCFD) determined by the product of the luminance and the area of the stimulus at the location of deployed attention triggers pupillary constriction.

## Materials and Methods

### Participants

About 20 healthy humans (13 men and 7 women) with correct or corrected to normal vision were recruited to perform a pair of choice-countermanding (CC) tasks. Their age ranged from 18 to 26 years, with an average (±SD) of 21 (±2.49) years. Participants received written and verbal instructions to perform the task in their preferred language either English or native Hindi, and they gave their consent to participate in this study in writing beforehand. They all were naive in performing these tasks and unaware of the objective of this study. This study was conducted in compliance with the Declaration of Helsinki (World Medical Association, 2013) and approved by the Institutional Ethics Review Board of University of Allahabad.

### Apparatus

Participants sat on a chair comfortably about 57 cm from a 19-inch LCD display monitor (resolution: 640 **×** 480, refresh rate: 60 Hz, and aspect ratio: 4:3) in an otherwise dark room. A custom-made chin-forehead resting apparatus was used to minimize the movements of a participant’s head. The center of the display and eyes were placed on a horizontal plane by adjusting the height of the chinrest, chair, and monitor. Participants got accustomed to the ambient darkness of the room during the adjustment of the sitting arrangements and recording apparatus, and to the task during a practice session (see the section “Estimation of Baseline Reaction Time”). A video-based desktop-mounted IR eye tracker interfaced with TEMPO-VideoSync software (Reflective Computing, St. Louis, MO, United States) in real time recorded the pupil area and gaze location at 240 Hz (Model: ETL-200; ISCAN Inc., Woburn, MA, United States). The spatial resolution (root mean square error) of the eye tracker was ∼0.1**°** visual angle.

### Tasks and Stimuli

The pupil dynamics were contrasted in the two experimental conditions of a variant of the CC task (Indrajeet and Ray, 2019; Middlebrooks et al., 2020). In both experiments, a decision cue was placed either at the center or periphery of the display monitor. Each subject randomly participated in both experiments.

## Experiment 1: Central Choice-Countermanding Task

**FIGURE 1:**
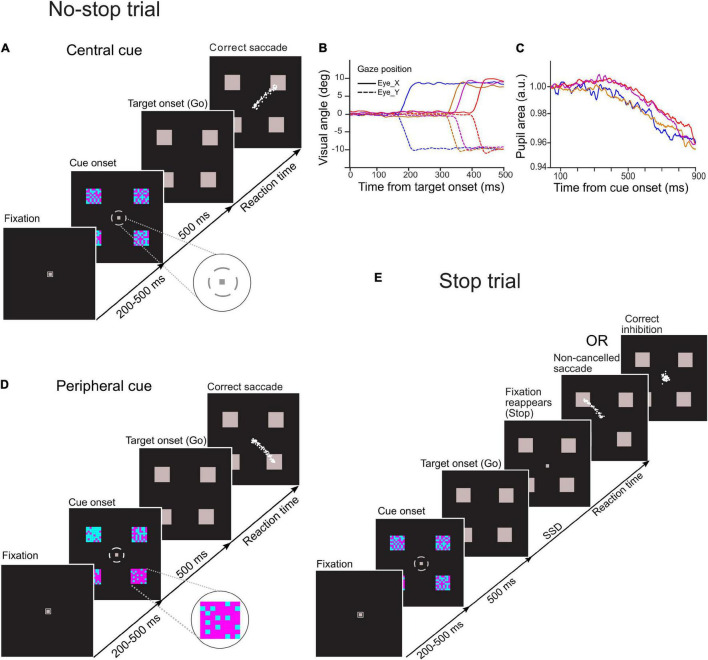
Schematic of temporal sequence of events in novel choice-countermanding (CC) tasks. Following the fixation duration, four cyan-magenta checker boxes appeared peripherally along with a small gray broken circle around the fixation spot. After 500 ms, the fixation spot and the broken circle disappeared, and all four peripheral checker boxes were masked by gray squares simultaneously (go-signal). **(A)** In the central cueing task, in the majority (60%) of trials (no-stop trials), participants were instructed to select the largest circumferential gap of broken circle (magnified in the inset) and to orient the gaze to one of the squares in the direction of the largest gap. **(D)** In the peripheral cueing task, in 60% of total trials (no-stop trials), participants were asked to select the checker box with the largest proportion of magenta color (magnified in the inset) and to orient their gaze to the location of the selected target following the disappearance of the fixation spot within a predetermined fixed period. Eye traces from representative saccades in no-stop trials are shown by white dots. **(E)** In the remaining trials (40%) of each task (stop trials), the fixation spot reappeared after a random delay [i.e., stop-signal delay (SSD)] to instruct participants to withhold their eye movements. No-stop and stop trials were randomly interleaved in each task. Gaze position and the pupillary response from representative trials are demonstrated in panels **(B,C)**, respectively.

A schematic of the temporal sequence of events in the task and behavior is shown in Figure 1A. Each trial began with the presentation of a gray fixation spot (∼0.25° × 0.25° visual angle) within a small gray square (∼0.5° × 0.5° visual angle) at the center of a monitor. After the participant maintained an uninterrupted gaze fixation for a period of 200–500 ms, the square enclosing the fixation spot disappeared, and four checker boxes (∼2.5° × 2.5° visual angle) appeared simultaneously at the periphery along with a broken gray circle of the outer and inner radius of ∼1.8° and ∼1.6° visual angles, respectively, around the central fixation spot. The obligatory fixation duration was randomized across the trials to reduce the influence of vigilance (i.e., non-selective attention in expectation of the onset of a decision cue), and the square enclosing the fixation spot at the foveal location was extinguished to reduce arousal induced by the cue onset on pupil size.

Each circumferential gap between the consecutive segments of the broken circle was aligned to a peripheral checker box. The difficulty in target selection was manipulated by changing the difference in dimensions among circumferential gaps of the broken circle. Each gap subtended an angle at the central fixation spot ranging either from 15° to 75° at an interval of 20°(easy task), or from 30° to 60° at an interval of 10° (difficult task). A total of 64 small squares of the same size arranged in eight rows and columns formed a square checker box, and they were painted in either cyan or magenta color. Each of these checker boxes was π/2 radian distant from any of its immediate adjacent checker box on an imaginary circle of radius of 12° visual angle with the origin at the center of the display monitor. An equal proportion of magenta- and cyan-colored squares was used to form each checker box. No specific pattern was followed either for the spatial distribution of the colored squares within any checker box, or for circumferential gaps in the broken circle (i.e., decision cue). Participants were asked to select the largest gap in the broken circle while maintaining their gaze at the fixation spot. After fixation for 500 ms, all checker boxes were masked by gray squares of the same dimensions, and both the fixation spot and the broken circle disappeared simultaneously. The disappearance of the fixation spot served as a *go-signal* for the elicitation of a saccade toward the gray square aligned with the selected gap in the broken circle.

## Experiment 2: Peripheral Choice-Countermanding Task

A schematic of the task is presented in Figure 1D. Overall stimulus quality and the temporal sequence of stimulus events in this task fundamentally remained the same as the central choice-countermanding (CCC) task described earlier, except that the proportions of cyan and magenta squares varied in four checker boxes, and each circumferential gap in the broken circle surrounding the fixation spot subtended 45° arc at the center of the display. The contrast between cyan and magenta colors in the checker boxes determined the difficulty of target selection. The proportions of magenta squares were distributed across four checker boxes either from 20 to 80% at an interval of 20% (easy trials), or from 40 to 70% at an interval of 10% (difficult trials). The colored squares within a checker box were not arranged in any specific spatial pattern. Their spatial distributions changed randomly in every trial. While maintaining their gaze at the fixation spot, participants were asked to assess the proportion of magenta color in each checker box. Both the broken circle and the fixation spot disappeared after 500 ms of viewing, and all checker boxes were masked by gray squares of the same dimensions. Subsequently, participants oriented their gaze toward one of the gray squares, which was previously occupied by a checker box with the highest proportion of magenta. The disappearance of the fixation spot served as a go-signal for the initiation of a saccadic eye movement to indicate their selection.

In a trial of any cueing conditions (central/peripheral), the four checker boxes appeared on an imaginary circle of radius of 12° visual angle either in a diamond formation or in a square formation. While the checker boxes in the diamond formation subtended 0, π/2, π, and 3π/2 radian angles, in the square formation they subtended π/4, 3π/4, 5π/4, and 7π/4 radian angles at the center with respect to the horizontal meridian of the screen. In each trial, one of these stimulus locations was randomly designated as the location of the potential saccade target. Subsequently, all checker boxes were masked by gray squares of the same dimensions. All four gray squares remained on the screen until the trial ended. In both the experiments, all stimuli were presented on a black background. The luminance of magenta, cyan, and gray (mask) color stimuli used in the task was, respectively, 3.844 cd/m^2^ (CYI: *X* = 0.186, *Y* = 0.239), 3.588 cd/m^2^ (CYI: *X* = 0.317, *Y* = 0.169), and 3.541 cd/m^2^ (CYI: *X* = 0.315, *Y* = 0.343), and that of the black background was 0.245 cd/m^2^ (CYI: *X* = 0.312, *Y* = 0.302).

### No-Stop and Stop Trials

In both experiments, participants were instructed to look at the selected target following the disappearance of the fixation spot (i.e., go-signal) unless the fixation spot reappears. In 60% of the total number of trials, we refer to “*no-stop trials*,” the fixation spot did not reappear (Figures 1A,D). In the remaining trials, we refer to “*stop trials*,” the fixation spot reappeared at a variable delay after the go-signal onset. It acted as a stop-signal instructing the participants to withhold their eye movements and maintain fixation (Figure 1E). The delay between go- and stop-signal, commonly known as “*stop-signal delay (SSD),”* randomly varied from 100 to 600 ms at an interval of 100 ms, with a jitter of about ± 8 ms (i.e., half of the screen refresh duration at the refresh rate of 60 Hz). No-stop and stop trials were randomly interleaved. An interval of 2,000 ms was introduced between consecutive trials.

The total number of trials performed by an individual varied between 400 and 415 in each experiment. In the rewarded no-stop trials, participants correctly selected the target and oriented the gaze to the target within the stipulated period. In the rewarded stop trials, participants successfully maintained the gaze at the central fixation spot for at least 300 ms after the stop-signal onset. The total number of correct no-stop and stop trials determined the monetary reward given to each participant. In each correct trial, participants received feedback on their performance through a 1,048-Hz auditory tone that lasted 200 ms.

### Estimation of Baseline Reaction Time

Prior to the main recording session, each participant was provided with 20–25 no-stop trials to estimate the baseline saccadic latency in the absence of stop-signal. All participants were entirely unaware of the stop-signal while performing these trials. The mean saccadic reaction time (RT) was calculated online after ∼15 correct trials, which was used as the baseline RT for the individual throughout the main recording session. The deadline for saccade elicitation was roughly 1.5 times the baseline RT rounded off to multiples of 100 ms, ranging from 400 to 700 ms. Participants were not instructed to make an eye movement to the target as quickly as possible. However, we pseudo-randomly interleaved no-stop and stop trials and set a fixed maximum allowed saccade RT to prevent them from waiting for the stop-signal.

### Procedure

In-house programs written in the Protocol Control Language (PCL) of TEMPO/VideoSYNC software displayed the stimuli, sampled and stored the eye position, pupil area, and other task contingencies in real time, and provided auditory feedback at the end of each correct trial. A virtual square electronic window (∼5° × 5°) around the targets specified the saccade-target region and another smaller window (∼4° × 4°) around the central fixation spot determined the gaze fixation region. All offline analyses were performed by using in-house programs written in Matlab ^®^ (The Mathworks, Inc., United States).

A box-car window filter of length 5 was applied to smoothen the horizontal and vertical components of the eye positions (Figure 1B). Subsequently, when the eye velocity and acceleration exceeded 30**°**/s and 300**°**/s^2^, respectively, an offline program demarcated the onset of a saccade, and an end of the saccade was demarcated when the eye velocity and deceleration decreased below the same criteria (Indrajeet and Ray, 2020). The demarcations of saccades at the beginning and end of each valid trial were further scrutinized manually through a visual inspection. Trials with blink-perturbed saccades were removed from subsequent analyses.

### Pupillometry

ISCAN eye tracker recorded pupil size in an arbitrary unit. Discontinuities and unusual task-irrelevant large modulations in the pupil data during fixation were observed in some trials, which might be due to blinks or partial occlusions of the eyes. We removed trials for analyses that exhibited a difference between the maximum and the minimum pupil size exceeding 20 × 10^3^. Pupil area was normalized by a divisive method (e.g., Park and McAnany, 2015; van Kempen et al., 2019; cf. Mathôt et al., 2018), where the average pupil area over the duration of 100 ms that spanned from 200 to 100 ms before the cue onset was used as the denominator. Subsequently, normalized pupil size in each trial was aligned either at the cue or saccade onset, smoothened by a box-car filter of length 5, and corrected to the baseline of 1.0 in an arbitrary unit. A discrete Fourier transformation on the pupil data in each trial generated a power spectrum of the signal. We removed outliers using the conventional interquartile range (IQR) method based on power at frequency of 0 Hz We contrasted the pupil dynamics relative to the onset of a decision cue. The line of sight moves away from the direction of camera after a gaze is shifted to the target. Because a circular disk appears to be elliptical when observed obliquely, the estimated pupil size using the eye tracker decreases immediately after a saccade, which is known as pupil-foreshortening error (PFE) (Gagl et al., 2011; Hayes and Petrov, 2016). We contrasted the pupil dynamics relative to the saccade onset as well to avoid PFE issues.

### Biomechanical Model of Pupillary Light Response

In order to test whether the eCFD (i.e., luminance multiplied by the area of the stimulus at the focus of attention), instead of the global luminance of the entire visual field, drives PLR, we simulated a homeomorphic biomechanical model of PLR (Usui and Hirata, 1995). As the model had no means to account for either an increase or a decrease in attentional breath (i.e., spatial extent of area over which attention is deployed, or scope of attention), mimicking the empirical data with the help of model simulation would also rule out an alternative explanation that mere changes in attentional scope due to a change in the location of the decision cue modulated pupillary constriction. This model was inspired by Hill’s muscle model (Hill, 1938; McMahon, 2020). In this model, inputs from the parasympathetic and sympathetic division of the autonomic nervous system (ANS) determined the non-linear interactions between the dynamic properties of sphincter and dilator iris muscles, respectively. A set of differential equations, as given in the Supplementary Material, were used to simulate the dynamics of pupillary aperture in response to light. In their framework, each pupillary muscle type was modeled by using an active contractile element (CE), a viscous element (VE), and a tension generator, based on physiological findings (Huxley and Niedergerke, 1954, 1958; Gordon et al., 1966). Peterson and Richmond (1988) noted that “Homeomorphic models are those whose elements correspond to the anatomical, physiological, biomechanical, and neural elements of the experimental system,” and “typically have more parameters than the less realistic phenomenological or input/output models.” In addition, the elements of a homeomorphic model rely on minimal assumptions, and computationally correspond to the morphology and physiology of an organism. Therefore, the falsification of a homeomorphic model is highly unlikely if parameterized properly.

We simulated the model for 100 trials each in central and peripheral cue conditions using Matlab Simulink ^®^ 8.6 (The Mathworks, Inc., United States) software running on an iMac (Apple Inc., United States) computer with a 3.2 GHz Intel ^®^ Core i5 processor, a 8 GB RAM, and OSX 10.11.4 operating system. The simulation of the model continued for 1,500 ms at an equal interval of 4 ms. Table 1 shows the parameters used for the simulation of the model.

**TABLE 1:** Parameters used for the simulation of a biomechanical model of pupillary light response (PLR).

Model parameter	Value	Description	Referring Supplementary Equations
α_*s*_	3.66	Off slope of isometric twitch of sphincter	20
α_*d*_	0.48	Off slope of isometric twitch of dilator	14
β_*s*_	8.12	On slope of isometric twitch of sphincter	20
β_*d*_	1.44	On slope of isometric twitch of dilator	14
*t*_*Ds*_	0.14	Visual delay to parasympathetic PLR pathway	20
*t*_*Dd*_	0.69	Visual delay to sympathetic PLR pathway	14
*a_s*	0.09	Passive tension coefficient for sphincter	15
*a_d*	0.72	Passive tension coefficient for dilator	9
*b_s*	0.36	Passive tension coefficient for sphincter	15
*b_d*	0.75	Passive tension coefficient for dilator	9
*c_s*	85.33	Elasticity coefficient for sphincter	17
*c_d*	15.17	Elasticity coefficient for dilator	11
*l*_*0s*_	1.49	Length of sphincter at rest	15
*l*_*0d*_	1.07	Length of dilator at rest	9
*L*_*0s*_	3.75	Length of sphincter at which *P*_*0s*_ is generated	17
*L*_*0d*_	4.58	Length of sphincter at which *P*_*0d*_ is generated	11
*x*	2.70	Initial radius of pupil	1, 4, 9, 11, 15, 17
*x*_*max*_	5.00	Maximum radius of pupil	9, 11
*P*_*0s*_	950.93	Maximum active tension in sphincter	17
*P*_*0d*_	119.41	Maximum active tension in dilator	11
*D*	13.18	Viscous coefficients at the phase of stretch	2, 22
*D*_−_	69.10	Viscous coefficients at the phase of release	2, 22
*E*_*sstat*_	0.05	Static component of parasympathetic activity	24, 25
*E*_*dstat*_	0.15	Static component of sympathetic activity	23, 25
E^s(t)	0.63	Dynamic component of parasympathetic autonomic activity	24, 26
E^d(t)	−0.54	Dynamic component of sympathetic autonomic activity	23, 26
λ	1	Gain	26
*V*_*in*_	CCP: 30 CCC: 20	Visual input (effective corneal flux density)	7, 14, 20

### Hypotheses and Statistical Analyses

Here, we tested the hypothesis that the strength of visual stimulation that drives pupillary response is determined by the eCFD, i.e., the luminance multiplied by the size of the object(s) at the location of attentional spotlight. To this end, we contrasted pupil size in the two task conditions, wherein the location of a decision cue was differed by its eccentricity and size but not the luminance. The stimuli and the temporal sequence of their appearance, hence the global CFD, were kept identical between the tasks. Since a pre-saccadic obligatory shift of attention occurs only when the elicitation of a saccade is inevitable (Born et al., 2014), in correct stop trials wherein the planned saccades were canceled, pre-saccadic attention presumably did not shift to the peripheral target. We further tested the hypothesis that a pre-saccadic obligatory shift of attention to the target location do not contribute to the PLR, by comparing pupil size in a subset of trials in both tasks when participants successfully inhibited the planned saccades.

SigmaStat (Systat, Inc., CA, United States) or Matlab ^®^ Statistical Toolbox (The Mathworks, Inc., United States) was used to perform all statistical computations. A sample size of 20 healthy participants who performed both tasks achieved a *post hoc* power of paired *t*-test comparison of the average pupil size at the time of saccade onset (i.e., when participants indicated their choice of target) being equal to 0.66 (G*Power software, version 3.1.9.7; Faul et al., 2009), and showed a significant difference between the task conditions (*p* = 0.02, *d* = 0.56). A Cohen’s *d* of 0.5 for within design suggests that the means of individuals’ differences between the two conditions differ by half the SD of the differences, which is interpreted as a moderate effect and statistically acceptable result (Cohen, 1988; Fritz et al., 2012). A moderate effect size might be due to the fact that the pupil data are inherently noisy (Kret and Sjak-Shie, 2019). We pooled several hundreds of normalized, smoothened, and baseline-corrected pupil size in all valid trials across all participants. Previously, the collated pupil data yielded a reliable comparison of the average pupil size, especially in the studies that recruited relatively fewer participants (*n* ≤ 20), and allowed fitting a unique model to the average PLR across trials (e.g., Mathôt et al., 2015; Pandey and Ray, 2021). In total, we pooled 3,531 trials (1,779 correct no-stop trials, 902 canceled stop trials, and 850 non-canceled stop trials) in Experiment 1, and 3,579 trials (1,900 no-stop trials, 803 canceled stop trials, and 876 non-canceled stop trials) in Experiment 2 for subsequent analyses.

## Results

### Performance and Reaction Time

**FIGURE 2:**
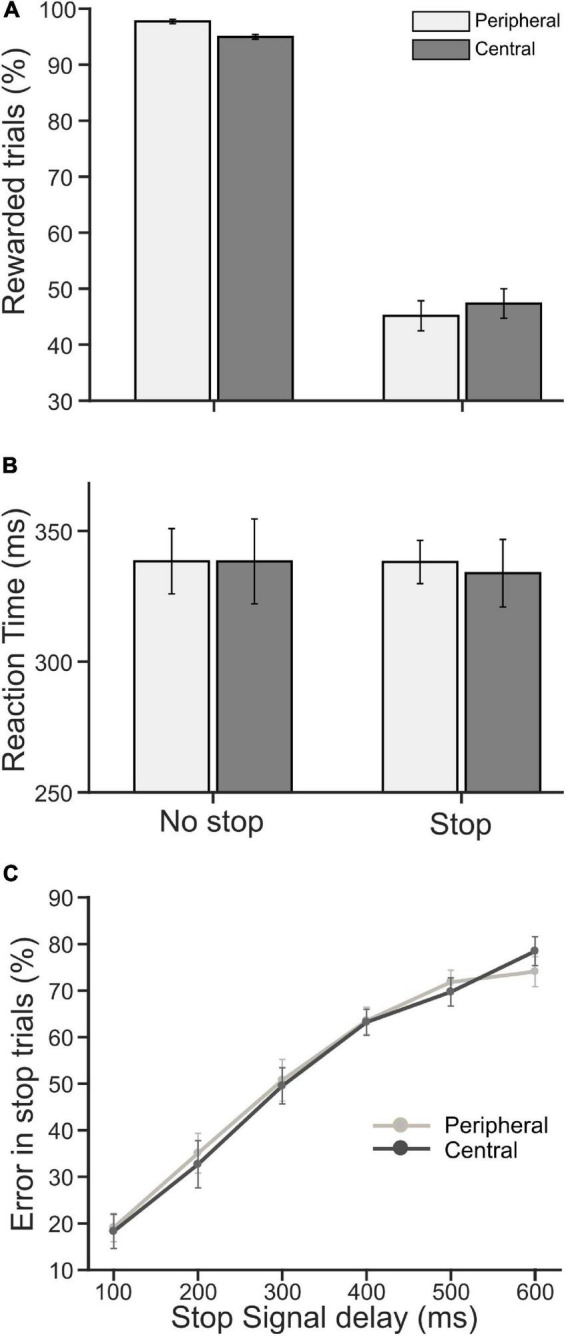
Population average of performance and reaction time (RT). **(A)** The average percentage of rewarded trials (i.e., correct no-stop and canceled stop trials) in peripheral (light gray) and central (dark gray) cueing for no-stop and stop conditions. **(B)** The average saccadic RT in correct no-stop and non-canceled stop trials in the peripheral (light gray) and central (dark gray) cueing task. **(C)** The average percentage of error/failure in stopping a saccade gradually increased as SSD increased in the peripheral (light gray) and central (dark gray) cueing task. Error bars indicate SE of corresponding mean.

Each participant performed a pair of CC tasks. The overall stimulus quality remained the same between the tasks, except the locations of a decision cue for the selection of the target were different—in one task, the cue appeared near the center of a display monitor [central choice countermanding (CCC task)], and in another, it appeared on the periphery [peripheral choice-countermanding (CCP) task]. However, in both tasks, the stop-signal appeared at the center (see Figure 1, and section “Materials and Methods” for details of the tasks). In these tasks, participants were required to either orient the gaze toward the selected target in the majority of trials, or refrain from the elicitation of a saccade in response to an infrequent stop-signal. First, we analyzed participants’ decision-making and countermanding behavior in both CCC and CCP tasks to examine whether they indeed followed the instructions given to perform the tasks. The average (±SEM) proportion of correct no-stop trials across the population of participants was 94.95 (±0.41) and 97.70 (±0.39)% in the CCC and CCP task, respectively. A paired *t*-test suggests that correct choice performance between the tasks was significantly [*t*(19) = 3.81, *p* = 0.0012, *d* = 0.85] different from each other for no-stop trials. The average (±SEM) percentage of successfully canceled stop trials was 47.4% (±2.63)in the CCC task and 45.20 (±2.67)% in the CCP task. However, no significant difference (*p* = 0.39) was found in the stopping performance between the tasks (Figure 2A). The average (±SEM) saccadic RT of correct no-stop trials across the population of participants was 338 (±16) and 338 (±12) ms for the CCC and CCP task, respectively. The average (±SEM) RT of a non-canceled stop trial for the CCC task was 334 (±13) ms, and for the CCP task that was 338 (±8) ms (Figure 2B). A paired *t*-test indicated no significant difference (*p* > 0.05) between the average RT in the CCC and CCP task for both correct no-stop and non-canceled stop trials. In Figure 2C, the average (±SEM) percentage of errors in stopping across the participants is plotted against SSD. A repeated measure two-way ANOVA indicated a monotonic increase in stopping errors with increasing SSD across the participants. The main effect of SSD on the percentage of failure in the inhibition of planned saccades (i.e., non-canceled stop trials) was found to be significant [*F*(5,95) = 91.859, *p* < 0.001], which was independent of the type of the task performed [*F*(1,95) = 0.0179, *p* = 0.895]. No significant effect of interaction (task type × SSD) was found [*F*(5,95) = 0.48, *p* = 0.789]. The Holm–Sidak method of *post hoc* pairwise multiple comparisons revealed that the percentage of non-canceled stop-trials in all possible SSD pairs between 100 and 400 ms was significantly different from each other (*t*_min_ = 3.904, *t*_max_ = 17.41, *p* < 0.001). These results indicate that participants carefully discriminated the target from the distractors irrespective of the placement of a decision cue at the parafoveal or perifoveal location, and deliberately attempted to inhibit a planned gaze shift toward the target in response to sudden appearance of the stop signal.

The average (±SEM) RT across pooled no-stop trials was 340 (±3) and 334 (±2) ms, in the CCC and CCP task, respectively. The average RT (±SEM) across pooled non-canceled stop trials was 324 (±4) and 331 (±3) ms, in CCC and CCP, respectively. The difference between the average RTs in the tasks was not significant, neither for no-stop trials (*p* = 0.14) nor for non-canceled stop trials (*p* = 0.21). Pupillary responses were compared in the two tasks for RT matched correct no-stop trials, and non-canceled stop trials separately.

### Pupillary Light Response

**FIGURE 3:**
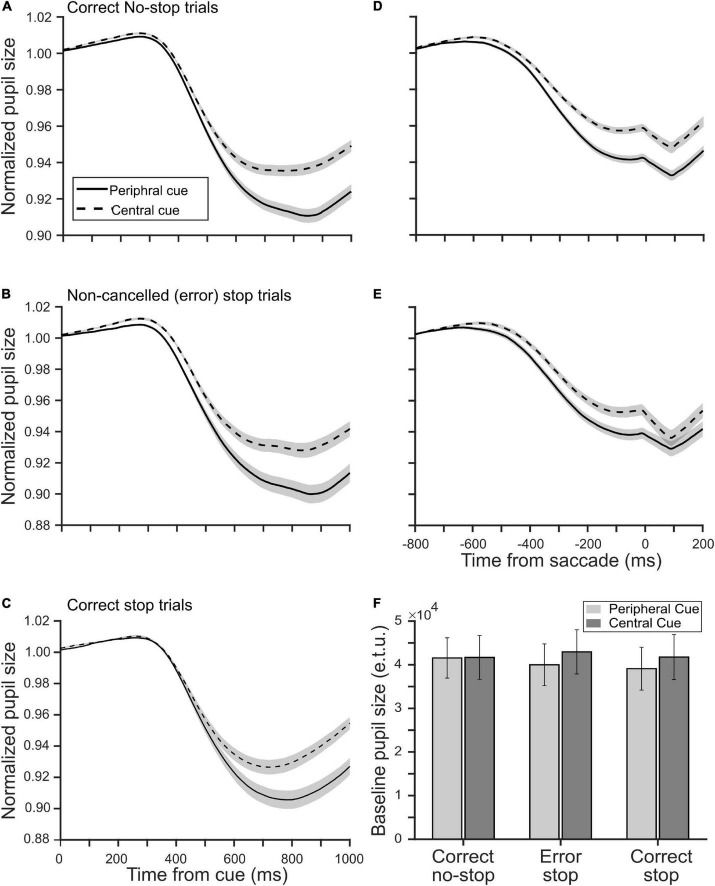
Influence of a decision cue on the pupil dynamics relative to the cue onset **(A–C)** and saccade onset **(D,E)** in the peripheral (solid) and central (dashed) cueing task. The smoothened, normalized, and baseline-corrected pupil size averaged across trials pooled from the population of 20 participants in panels **(A,D)** the correct no-stop, **(B,E)** non-canceled stop, and **(C)** correct stop condition. The gray patches are overlaid on the traces to show corresponding SE of the mean pupil size. **(F)** The population average of tonic pre-stimulus pupil size in the eye tracker’s unit during the fixation period in correct no-stop, non-canceled stop, and correct stop trials in the peripheral (light gray) and central (dark gray) cueing task.

Next, we sought to know if the location of a decision cue affected pupillary aperture, especially when it was placed at the periphery where vision was impoverished. Any significant difference in the pupillary dynamics between the tasks would suggest some kinds of non-trivial pupillary light reflex (PLR), which was not merely driven by differential CFD, because the global luminance of the stimuli in both the tasks remained the same. All trials were segregated as correct no-stop, successful stop, and non-canceled stop trials for both CCC and CCP tasks. Figures 3A,B show a modulation in the average (±SEM) normalized and baseline-corrected pupil size following the cue onset across all correct no-stop trials and non-canceled stop trials for both the tasks, respectively. We performed a two-tailed *t*-test to contrast the two independent means between normalized baseline-corrected pupil area in the CCC and CCP task sampled at every 4 ms for a span of a second from the cue onset. In correct no-stop and non-canceled stop trials, the pupil constricted significantly more (*p* < 0.05) in the CCP task than in the CCC task starting from 420 to 372 ms after the decision cue onset, respectively. We calculated the magnitude of the maximum pupillary constriction by subtracting the minimum pupil size in each trial from baseline 1. Mann–Whitney rank sum test showed that the median magnitude of the maximum pupillary constriction across correct no-stop trials (CCP: 4.3%, CCC: 3.1%, *U* = 13.58 × 10^5^) and non-canceled stop trials (CCP: 4.6%, CCC: 3.5%, *U* = 31.28 × 10^4^) was significantly (*p* < 0.001) higher in the CCP task than in the CCC task.

Because of variable RT across trials, the abovementioned analysis cannot guarantee that the maximum constriction happened always before saccade onset. To contrast the pupil dynamics strictly before gaze orientation and avoid the pupil-foreshortening error (see the section “Materials and Methods”), we aligned the normalized and baseline-corrected pupil area on saccade onset to contrast the magnitude of pupillary constriction between the tasks (Figures 3D,E). Mann–Whitney rank sum test between the tasks for both no-stop and non-canceled stop trials indicates that the median magnitude of pupillary constriction at the moment, when subjects indicated their judgment about the location of the target by initiating gaze orientation, was significantly higher (*p* < 0.001) in the CCP task than in the CCC task in correct no-stop (CCP: 5.5%, CCC: 3.9%, *U* = 11.84 × 10^5^) and non-canceled stop (CCP: 3.2%, CCC: 1.5%, *U* = 26.37 × 10^4^) trials.

In the CCP task, covert selective attention was distributed and deployed toward the periphery to discriminate the target from distractors; in contrast, attention was presumably concentrated near the central fixation spot in the CCC task. To test whether spatially congruent selective attention and pre-saccadic attention together resulted in more constriction in the CCP task as shown in Figures 3A,B, we contrasted pupil size in correct stop trials in both tasks (Figure 3C). Any difference in the pupil dynamics in correct stop trials between the tasks would suggest differential effect of the eccentricity of a decision cue and/or attentional breadth, but not pre-saccadic orientation of attention, on pupil size. We performed a two-tailed *t*-test to contrast the two independent means between normalized baseline-corrected pupil area in the CCC and CCP task sampled at every 4 ms for a span of a second from cue onset. In correct stop trials, the pupil constricted significantly more (*p* < 0.05) in the CCP task than in the CCC task starting from 552 ms after the cue onset. Mann–Whitney rank sum test showed that the median magnitude of constriction was significantly (*p* < 0.001, *U* = 30.41 × 10^4^) higher in the CCP task than in the CCC task in correct no-stop trials (CCP: 4.0% and CCC: 3.1%).

Further, we tested whether the pre-saccadic obligatory orientation of attention played any role at all in controlling pupil size. We considered a subset of non-canceled trials wherein fixation was maintained for at least 800 ms following a decision cue (i.e., 300 ms following the target onset/go-signal). This was because around that time pupils constricted to a maximum (Figures 3B,C). Note that the average RT relative to the target onset/go-signal across the population of participants in non-canceled stop trials for the CCC and CCP task was 334 and 338 ms, respectively. Thus, a total of 472 non-canceled stop trials in the CCC task and 520 non-canceled stop trials in the CCP task, which yielded *RT* ≥ 300 ms, contributed data to contrast between pupil size when saccades were canceled vs. when elicited in stop trials for each task. Mann–Whitney rank sum test showed that the median of averaged normalized pupil size in a span of 800 ms from the cue onset across non-canceled and canceled stop trials were not significantly different either in the CCC task (non-canceled: 0.993, canceled: 0.992, *p* = 0.073) or in the CCP task (non-canceled: 0.990, canceled: 0.991, *p* = 0.37). Recall that pre-saccadic orientation of attention happens only if a saccade is certain (Born et al., 2014). This result, therefore, suggests that pre-saccadic obligatory attentional orientation did not play a critical role in PLR.

Raw pupil size was normalized by dividing it by the baseline pupil size. Any difference in the tonic pre-stimulus pupil size between the tasks within a type of trials therefore could yield false results. We compared the average raw pupil size during the fixation period of 300 ms before the cue onset for each participant and each type of trials between the tasks (Figure 3F). The average (±SEM) tonic pre-stimulus pupil size in an eye tracker’s unit for CCC and CCP was 41,669 (±5,003) and 41,553 (±4,622) in correct no-stop trials, 42,951 (±5,061) and 39,983 (±4,790) in erroneous (i.e., non-canceled) stop trials, and 41,767 (±5,159) and 39,094 (±4,906) in correct (i.e., canceled) stop trials, respectively. A paired *t*-test across the population of participants showed no significant (*p* > 0.05) difference.

### Model Simulation

**FIGURE 4:**
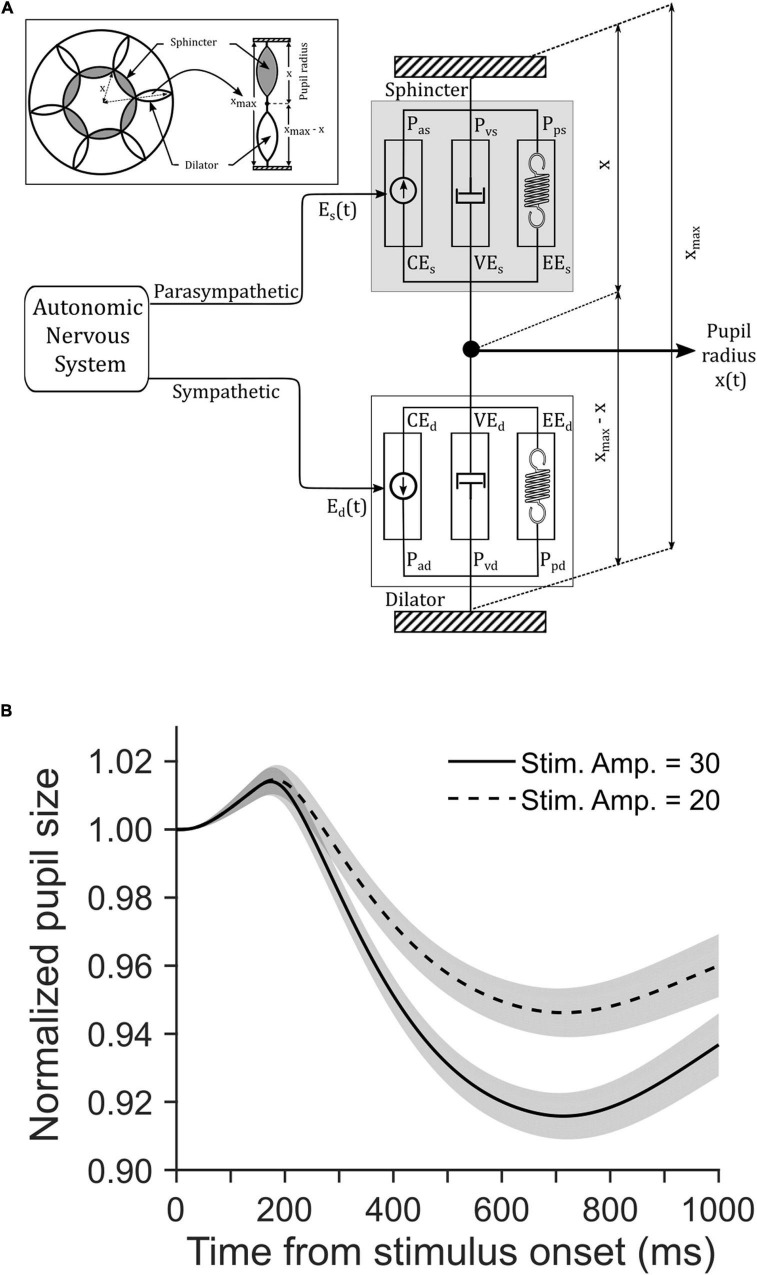
**(A)** A schematic of Usui and Hirata (1995) model of pupillary muscle plants. The model determined the non-linear interactions between the dynamic properties of sphincter and dilator muscle components as they received inputs from the parasympathetic and sympathetic division of the autonomic nervous system (ANS), respectively. Refer the Supplementary Material and Table 1 for details of parameters. **(B)** The influence of stimulus strength on simulated pupil dynamics from stimulus onset. Simulation of a biomechanical model of pupillary aperture (Usui and Hirata, 1995) with the two different stimulus strengths yielded stimulus amplitudes of 30 (solid) and 20 (dashed). Simulated pupil dynamics mimic the behavioral data (see Figures 3A–C). The gray patches are overlaid on the traces to show corresponding SE of the mean pupil size.

We simulated a biomechanical model of pupillary muscle plants (Figure 4A). In this homeomorphic model of PLR (Usui and Hirata, 1995), we used different strengths of visual stimulation for the two tasks. To this end, we defined a parameter *V*_*in*_ in the model (see Eqs 7, 14, 20 in the Supplementary Material). Other parameters of this model accounting for the mechanical properties of sphincter and dilator muscles, and the dynamics of the parasympathetic and sympathetic activity driving these muscles, respectively, remained the same between the tasks (Table 1). Figure 4B shows the average (±SEM) simulated pupil size across 100 trials in each task aligned on the cue onset. Mann–Whitney rank sum test showed that the median magnitude of constriction in the CCP task was significantly (*p* = 0.009, *U* = 3,929.5) higher than the CCC task in simulated trials (CCP: 10% and CCC: 7%). The resemblance between simulated and empirical pupil dynamics (comparing Figure 4B with Figure 3A) suggests that CFD determined by the global luminance of the entire visual field does not drive PLR, rather eCFD determined by the luminance of the stimulus within the area where covert selective attention is deployed does. Note that our goal was not to recreate the observed data *via* model simulation, rather to capture the trend of pupil dynamics in the two tasks with different stimulus strengths. Hence, the model parameters were not optimized; instead, we used arbitrary values within a physiologically feasible range.

## Discussion

In the current study, CC tasks enforced focused and diffused attention to discriminate the target from distractors, unlike previous studies wherein subjects narrowed or broaden attentional breadth during passive viewing, counting objects, or feature detection. We presented a decision cue either just outside the central vision (CCC task) or on the peripheral visual field (CCP task). Our data shows that pupil size decreased more when a decision cue appeared at the periphery than at the center, despite the same global luminance in both conditions, in all three behavioral conditions—correct no-stop, correct stop (i.e., canceled saccade), and incorrect stop (i.e., non-canceled saccade). Not only the luminance of the stimulus but also the size of the stimulus play a crucial role in the modulations of PLR: pupil size decreases with increasing CFD, which is the product of the luminance multiplied by the adapting field size of the stimulus (Moon and Spencer, 1944; Stanley and Davies, 1995; Park and McAnany, 2015; Hu et al., 2020). No change in the pupil diameter as a function of the size of the stimulus was observed when CFD remained fixed (Atchison et al., 2011). To explain our findings, we assumed an eCFD (i.e., the luminance multiplied by the area of the stimulus at the focus of covert attention) was higher in the CCP than in the CCC task. Here, we speculate that the CFD is not just a physical quantity defined by the product of luminance and area of all stimuli comprising the entire visual space, rather the luminance and area of stimuli where attention is deployed determines eCFD. In the current context, eCFD in the CCP task was determined using a decision cue in the form of an array of square checker boxes (each of size ∼2.5° × 2.5° at an eccentricity of ∼12°), whereas in the CCC task, a thin broken annular decision cue with a small outer (∼1.8°) and inner (∼1.6°) radius determined eCFD. We simulated a biomechanical model of pupillary aperture (Usui and Hirata, 1995; Pandey and Ray, 2021) with the two different stimulus strengths in an arbitrary unit, which we referred to as an effective CFD or eCFD. Simulated data mimicked the observed data, in support of the possibility of a critical contribution from selective attention in modulating pupil size. This adequately explains why the parafoveal and perifoveal decision cue differently contributed to pupillary constriction despite the same global luminance in the two tasks.

In the centrally cued condition (CCC) task of our experiment, a decision cue and the stop-signal both appeared in proximity at the parafoveal and foveal space, respectively, whereas in the peripherally cued condition (CCP) task, a decision cue and the stop-signal were quite apart on the display. We expected better stopping performance due to attentional facilitation in the central-cued condition. However, in contrast to our expectation, we did not observe an improvement in stopping performance in the central-cued condition. This happened possibly because the attention for visual selection/perceptual decision-making and also for action control are dissociable (Sharika et al., 2009). Even when the former moved to the periphery in the CCP task, the latter likely remained latched to the center of the display during fixation, in both the tasks. Middlebrooks and Schall (2014) also found that perceptual decision-making and control of action are independent processes, and perceptual decision-making had no influence on the ability to stop an impending saccade.

We dissociated the contributions of pre-saccadic attention from selective attention to PLR by contrasting the pupil dynamics in canceled and non-canceled stop trials. Although the coupling between attention and saccades is robust (Hoffman and Subramaniam, 1995; Kowler et al., 1995), it has been shown previously that attention does not shift to the target location if the planned saccade is canceled (Ignashchenkova et al., 2004; Ray et al., 2009; Born et al., 2014). Furthermore, recent studies questioned whether the observed facilitation in visual discrimination at the location of an imminent saccade was due to pre-saccadic orientation of attention or by means of the consolidation of visual short-term memory following the orientation of gaze (Born et al., 2013; Huber-Huber et al., 2021). Our data suggest that pre-saccadic orientation of attention did not contribute to the modulation of visually evoked pupillary responses, suggesting no high-level perceptual benefit from pre-saccadic pupillary constriction except the prevention from overwhelming visual transients on the fovea during the gaze-fixation-gaze cycle in a naturally heterogenous visual world (Mostofi et al., 2020; Pandey and Ray, 2021).

Whether the observed difference in the magnitude of pupillary constriction between the two experiments was due to the different visual strengths filtered by the scope of attention, or due to the disparity between the task conditions (i.e., finding the largest dimension in the central cue, vs. finding a maximum proportion of a color in the peripheral cue). Task-induced modulation in pupillary response was observed when participants were asked to identify either the larger (global) letter or the smaller (local) letter that constructed the larger letter (DiCriscio et al., 2018). In this task, participants might have also required to adjust attentional breadth from broad (global) to narrow (local) according to the demand of the task. In addition, unlike the current study, participants in their study exhibited different baseline pupil sizes and RTs, indicating the different levels of arousal and difficulty between the two task conditions. Previous studies have shown the effect of arousal (Bradshaw, 1967) and effort (Kahneman and Beatty, 1967) on a change in pupillary aperture. Previous imaging studies have shown that spatial attention affects the activity of V1 (Gandhi et al., 1999; Posner and Gilbert, 1999; Kelly et al., 2008; Hembrook-Short et al., 2019). An endogenous attentional signal for the detection of either the central or peripheral stimulus differentially modulated V1 activity. Recently, electrophysiology on non-human primates has confirmed the influence of attention even earlier at the lateral geniculate nucleus (LGN) (Shah et al., 2021). Visual stimulus in the blind field of a human subject with putative loss of unilateral striate cortex, and monkeys with unilateral damage in the primary visual cortex resulted in changes in pupillary aperture (Weiskrantz et al., 1998), indicating that the early visual system, which is susceptible to attentional modulation, plays a role in pupillary response. On the other hand, the discrimination between task rules occurs at relatively higher levels in the hierarchy of the decision-making and action planning pathways in the cortex; for instance, premotor, prefrontal, or supplementary motor areas (Wallis and Miller, 2003; Mian et al., 2014; Ray and Heinen, 2015), which do not have known direct projections to the PLR pathway (Wang and Munoz, 2015; Pandey and Ray, 2021). Furthermore, decision rule discrimination is a slow process, for instance, neurons in the supplementary eye field (SEF) consumes 250–600 ms after the decision-cue onset to discriminate decision rules based on the difficulty of the task (Ray and Heinen, 2015). In our current study, the pupil constricted significantly more in the peripherally cued task than in the centrally cued task between 370 and 420 ms after the cue onset. Given that a reflexive change in pupillary aperture in response to visual afferents is naturally slow with a delay of between 200 and 500 ms (Ellis, 1981), it appears that the observed differential pupillary constriction between the two experiments was more likely to be visually evoked than task-induced.

The eyes suffer from spherical and chromatic aberrations (Liang and Williams, 1997). Any change in pupil size modifies the eyes’ optics, which in turn influences the visual acuity (Campbell and Gregory, 1960). While the former causes blurred edge of an image due to unequal bending of light passing through the different parts of the lens, the latter creates a rainbow effect due to unequal bending of light with different wavelengths (Campbell and Gubisch, 1967; Charman et al., 1978). Involuntary constriction of pupil or miosis is a remedy to reduce aberrations by limiting incoming light through the edge of the eye lens. Pupillary constriction also increases the depth of field, which is equivalent to decreasing the focal length of the lens by means of accommodation reflex (Levin et al., 2011). A decrease in focal length (*f*) increases angular field of view (AFV) given fixed horizontal dimension (*d*) of the sensor (i.e., retina) following the formula AFV = 2tan^–1^ (*d*/2*f*) (Duchowski, 2017). In fact, retinal luminance is critical for eliciting accommodation reflex (Campbell, 1954; Kruger and Pola, 1986). Thus, pupillary constriction does not just regulate an influx of light, it improves the overall quality of the visual input by reducing aberrations, increasing the depth of field, and widening the view field. Woodhouse (1975) suggested that a constricted pupil could improve the visual acuity by a magnitude of about 20% (in specific situations) compared to a dilated pupil. In our experiment, when a decision cue appeared at the periphery where the visual acuity was mostly limited by the reduced density of cone photoreceptors, the pupillary response might automatically correct the spherical aberration induced by the task demands and assumed an optimal pupil size for the task (Mathôt and Ivanov, 2019). We observed more pupillary constriction in this task that required a larger view field for the discrimination of peripheral chromatic stimuli, suggesting that task-demand could modulate PLR for better visual perception.

## Data Availability Statement

The original contributions presented in the study are included in the article/Supplementary Material, further inquiries can be directed to the corresponding author.

## Ethics Statement

The studies involving human participants were reviewed and approved by Institutional Ethics Review Board of University of Allahabad. The patients/participants provided their written informed consent to participate in this study.

## Author Contributions

PP collected and analyzed data and performed statistics. SR designed and programmed the tasks, and programmed and simulated the model. Both authors wrote the manuscript.

## Conflict of Interest

The authors declare that the research was conducted in the absence of any commercial or financial relationships that could be construed as a potential conflict of interest.

## Publisher’s Note

All claims expressed in this article are solely those of the authors and do not necessarily represent those of their affiliated organizations, or those of the publisher, the editors and the reviewers. Any product that may be evaluated in this article, or claim that may be made by its manufacturer, is not guaranteed or endorsed by the publisher.
