# Supplementary material for: Influence of the Location of a Decision Cue on the Dynamics of Pupillary Light Response
Source: Front Hum Neurosci. 2022 Jan 26;15:755383. doi: 10.3389/fnhum.2021.755383 (PMC8826249; doi:10.3389/fnhum.2021.755383)
Supplement: Supplementary file 1 [file Data_Sheet_1.docx]

**SUPPLEMENTARY MATERIALS**

Hill’s model of skeletal muscle uses three-elements, a contractile element (CE), and two non-linear spring like elastic elements (EE) – one connected in parallel to CE and the other in series (Hill, 1938). In this model, active force in CE is generated by movements of cross-bridges between actin and myosin in sarcomere. When activated CE shortens in length. Proportional to the velocity of contraction from its resting length, a resistive force is generated in muscle, which causes damping and acts like a shock absorber. Passive tension is generated in EEs that represent connecting tissues running parallel to muscle fibers (e.g., sarcolemma, epimysium, perimysium, endomysium) and in series in tendon that fuses muscle to bone. In their model of pupillary light reflex (PLR), Usui and Hirata (1995) simplified the two-dimensional structures into a one-dimensional push-pull structure of iris muscles. They used an active contractile element (CE), a viscous element (VE), and an elastic element (EE) connected in parallel to model both constrictor and dilator muscle, but different parameters were used to characterize mechanical properties (Figure 4A). The VE in the model accounted for damping. The parasympathetic and sympathetic division of the autonomic nervous system (ANS) sent out projection to determine dynamic properties of sphincter and dilator muscle, respectively. The following general equations described the elasticity, viscosity and tension characteristics of pupillary muscles (Usui and Hirata, 1995; Yamaji et al., 2000; Pandey and Ray, 2021).

*Elasticity*:

$P_{p}=\left\{ \begin{matrix} a\left( x-l_{0} \right)^{4}+b\left( x-l_{0} \right)^{2},\left( x\geq l_{0} \right) \\ 0, \left( x<l_{0} \right) \end{matrix} \right.$ ………………………(1)

where *x* is the pupil radius, $a$ and $b$ are constants, and $l_{0}$ is the length of muscle at the rest.

*Viscosity*:

$P_{v}=\left\{ \begin{aligned} D_{+}\frac{dx}{dt},\left( \frac{dx}{dt}\geq0 \right) \\ D_{-}\frac{dx}{dt},\left( \frac{dx}{dt}<0 \right) \end{aligned} \right.$ ……………………………..(2)

where D_+_ and D_–_ are the viscous coefficients at the phase of stretch and release, respectively.

*Tension generator*:
$P_{a}=g\left( t \right)\cdot p_{a}(x)$ ……………………..(3)
$p_{a}\left( x \right)=\left\{ \begin{aligned} P_{0}-c\left( x-L_{0} \right)^{2},(P_{0}\geq c\left( x-L_{0} \right)^{2}) \\ 0, (P_{0}<c\left( x-L_{0} \right)^{2}) \end{aligned} \right.$ ……………………..(4)
$g\left( t \right)=g_{stat}+\overset{^}{g}(t)$ ……………………...(5)
$g_{stat}=\gamma\cdot E_{stat}$ …………………………..(6)
$\frac{d^{2}\overset{^}{g}(t)}{dt^{2}}+\left( 2\alpha+\beta\right)\frac{d\overset{^}{g}\left( t \right)}{dt}+\alpha\left( \alpha+\beta\right)\overset{^}{g}\left( t \right)=V_{in}\beta\overset{^}{E}(t-t_{D})$ …………...(7)

where $g\left( t \right)$and $p_{a}\left( x \right)$ are input dependent and muscle length dependent terms of active tension, respectively; *L_0_* is the muscle length at which maximum active tension *P_0_* is generated, respectively; $g_{stat}$ is the DC part of $g\left( t \right)$, while $\overset{^}{g}(t)$ represents the AC part; similarly, E_stat_ is the DC part of autonomic activity *E(t)* and *Ê(t)* is the AC part; *t_D_* is the delay time of response; *c* and *γ* are constants; *α* and *β* are the time constants of the off and on slope of the isometric twitch response, respectively. The parameter *V_in_* indicates visual strength or effective flux density (eCFD) that drives pupillary response, which assumed either 30 (CCP task) or 20 (CCC task) in arbitrary unit during simulation. In the following equations, subscript ‘s’ denotes sphincter parameters, subscript ‘d’ denotes dilator parameters, ‘stat’ and ‘^’ stand for static and dynamic part of the parameter; $x_{max}$ denotes the maximum radius of pupil.

System equation of the model:

$m\frac{d^{2}x}{dt^{2}}=T_{d}-T_{s}=P_{pd}+P_{ad}-P_{ps}-P_{as}-P_{v}$ ………(8)

**Dilator tension characteristics**:

*Passive tension*

$P_{pd}=\left\{ \begin{aligned} a_{d}\left( x_{max}-x-l_{0d} \right)^{4}+b_{d}\left( x_{max}-x-l_{0d} \right)^{2},(x_{max}-x\geq l_{0d}) \\ 0, (x_{max}-x<l_{0d}) \end{aligned} \right.$

………..(9)

*Active tension*

$P_{ad}=\left\{ \begin{aligned} g_{d}\left( t \right){\cdot p}_{ad}\left( x \right),(p_{ad}\left( x \right)\geq0) \\ 0, (p_{ad}\left( x \right)<0) \end{aligned} \right.$ …………(10)

*Elasticity*

$p_{ad}\left( x \right)=P_{0d}-c_{d}\left( x_{max}-x-L_{0d} \right)^{2}$ …………..(11)

*Dynamics*

$g_{d}(t)=g_{dstat}+{\overset{^}{g}}_{d}(t)$ ………….(12)

$g_{dstat}=\gamma_{d}E_{dstat}, (\gamma_{d}=1)$ ………….(13)

$$\frac{d^{2}{\overset{^}{g}}_{d}}{dt^{2}}+\left( 2\alpha_{d}+\beta_{d} \right)\frac{d{\overset{^}{g}}_{d}}{dt}+\alpha_{d}\left( \alpha_{d}+\beta_{d} \right){\overset{^}{g}}_{d}={V_{in}\beta}_{d}{\overset{^}{E}}_{d}(t-t_{Dd})$$

……..……(14)

**Sphincter tension characteristics**:

*Passive tension*

$P_{ps}=\left\{ \begin{aligned} a_{s}\left( x-l_{0s} \right)^{4}+b_{s}\left( x-l_{0s} \right)^{2},(x\geq l_{0s}) \\ 0, (x<l_{0s}) \end{aligned} \right.$ ………….(15)

*Active tension*

$P_{as}=\left\{ \begin{aligned} g_{s}\left( t \right)\cdot p_{as}\left( x \right),(p_{as}\left( x \right)\geq0) \\ 0, (p_{as}\left( x \right)<0) \end{aligned} \right.$ ………….(16)

*Elasticity*

$p_{as}\left( x \right)=P_{0s}-c_{s}\left( x-L_{0s} \right)^{2}$ ………….(17)

*Dynamics*

$g_{s}(t)=g_{sstat}+{\overset{^}{g}}_{s}(t)$ ………….(18)

$g_{sstat}=\gamma_{s}E_{sstat}, (\gamma_{s}=1)$ ………….(19)

$\frac{d^{2}{\overset{^}{g}}_{s}}{dt^{2}}+\left( 2\alpha_{s}+\beta_{s} \right)\frac{d{\overset{^}{g}}_{s}}{dt}+\alpha_{s}\left( \alpha_{s}+\beta_{s} \right){\overset{^}{g}}_{s}={V_{in}\beta}_{s}{\overset{^}{E}}_{s}(t-t_{Ds})$

………….(20)

Total viscosity of the sphincter and dilator

$P_{v}=P_{vd}+P_{vs}$ ………….(21)

$=\left\{ \begin{aligned} \left( D_{s+}+D_{d-} \right)\frac{dx}{dt}=D_{+}\frac{dx}{dt},(\frac{dx}{dt}\geq0) \\ \left( D_{s-}+D_{d+} \right)\frac{dx}{dt}=D_{-}\frac{dx}{dt},(\frac{dx}{dt}<0) \end{aligned} \right.$ ………….(22)

**Autonomic Nervous innervation**:

*Sympathetic nervous activity*

$E_{d}\left( t \right)=E_{dstat}+{\overset{^}{E}}_{d}\left( t \right),E_{d}\left( t \right)\geq0$ ………….(23)

*Parasympathetic nervous activity*

$E_{s}\left( t \right)=E_{sstat}+{\overset{^}{E}}_{s}\left( t \right),E_{s}\left( t \right)\geq0$ ………….(24)

Reciprocal relationship between the parasympathetic and sympathetic nervous activities

$E_{dstat}=\lambda_{stat}\left( 1-E_{sstat} \right), \lambda_{stat}=1, 0\leq E_{sstat}\leq1$ ………….(25)

${\overset{^}{E}}_{d}\left( t \right)=-\lambda{\overset{^}{E}}_{s}\left( t \right), 0\leq\lambda\leq1$ ………….(26)
